# Trends and clinicopathological predictors of axillary evaluation in ductal carcinoma in situ patients treated with breast‐conserving therapy

**DOI:** 10.1002/cam4.1252

**Published:** 2017-12-22

**Authors:** Nai‐si Huang, Jing Si, Ben‐long Yang, Chen‐lian Quan, Jia‐jian Chen, Jiong Wu

**Affiliations:** ^1^ Department of Breast Surgery Fudan University Shanghai Cancer Center No. 270, Dongan Rd Shanghai 200032 China; ^2^ Department of Oncology Fudan University Shanghai Medical College Shanghai China; ^3^ Collaborative Innovation Center for Cancer Medicine Shanghai China

**Keywords:** Axillary evaluation, breast‐conserving therapy, Ductal carcinoma in situ, sentinel lymph node biopsy

## Abstract

The aim of this study was to investigate the trends of axillary lymph node evaluation in ductal carcinoma in situ (DCIS) patients treated with breast‐conserving therapy (BCT) and to identify the clinicopathological predictors of axillary evaluation. DCIS patients treated with BCT in 2006–2015 at our institute were retrospectively included in the analysis. Patients were categorized into three groups: sentinel lymph node biopsy (SLNB), axillary lymph node dissection (ALND), and non‐evaluation. Univariate and multivariate logistic regression analyses were performed to identify factors that predicted axillary evaluation. A total of 315 patients were identified, among whom 135 underwent SLNB, and 15 underwent ALND. The proportion of patients who underwent axillary evaluation increased from 33.0% in 2006–2010 to 53.8% in 2011–2015 (*P *<* *0.001), however, no patients had lymph node metastasis based on final pathology. In multivariate analysis, high‐grade tumor favored axillary evaluation (OR = 4.376, 95% CI:1.410–13.586, *P *=* *0.011); while excision biopsy favored no axillary evaluation compared with other biopsy methods (OR = 0.418, 95% CI: 0.192–0.909, *P *=* *0.028). Subgroup analysis of patients treated in 2011–2015 revealed that high‐grade tumor (OR = 5.898, 95% CI: 1.626–21.390, *P *=* *0.007) and palpable breast lump (OR = 2.497, 95% CI: 1.037–6.011, *P *=* *0.041) were independent predictors of axillary lymph node evaluation. Despite the significant decrease in ALND and a concerning overuse of SLNB, we identified no axillary lymph node metastasis, which justified omitting axillary evaluation in these patients. High‐grade tumor, palpable lump, and biopsy method were independent predictors of axillary evaluations. Excision biopsy of suspicious DCIS lesions may potentially preclude the invasive component of the disease and help to avoid axillary surgery

## Introduction

Ductal carcinoma in situ (DCIS) constitutes approximately 20% of all newly diagnosed breast cancers due to increased screening and improvements in the sensitivity of mammography 1. Pure DCIS is a noninvasive local disease, with little propensity for lymphatic metastases 2, 3. The risk of death from breast cancer is as low as 2% within the 10 years following the diagnosis of DCIS 4. Therefore, surgical excision is the major treatment strategy for DCIS.

The treatment options for DCIS are breast‐conserving therapy (BCT) or mastectomy, depending on the extent and grade of the DCIS lesion, patient preference, and other risk factors 5. Recent data indicated that 67%–90% of DCIS patients worldwide have been treated with BCT 6, 7. Unlike invasive breast cancer, there is no demonstrated benefit of axillary lymph node evaluation in DCIS cases 8, 9. According to the current recommendations of the National Comprehensive Cancer Network (NCCN) and American Society of Clinical Oncology (ASCO), axillary lymph node dissection (ALND) should not be performed in the absence of evidence of invasive cancer in DCIS patients. Sentinel lymph node biopsy (SLNB) is justified in mastectomy patients because mastectomy may preclude future SLNB if invasive cancer is discovered. For patients treated with BCT, SLNB can be considered in the following scenarios: (1) palpable mass at physical examination; (2) suspicion of invasive cancer on imaging; (3) DCIS area larger than 5 cm; and (4) surgical excision at an anatomical location that would preclude future SLNB 8, 10.

The aim of the current study was to investigate the trends in axillary lymph node evaluation in DCIS patients treated with BCT in China and to identify the clinical, radiological, and pathological predictors of axillary evaluation.

## Materials and Methods

### Patients

We retrospectively reviewed the medical records of DCIS patients who underwent surgery from 2006 to 2015 at Fudan University Shanghai Cancer Center (FUSCC). The following inclusion criteria were applied: (1) patients were diagnosed with pure DCIS; (2) patients were treated with BCT; and (3) DCIS was unilateral. Patients were excluded if they had the following: (1) invasive disease, including micro‐invasion; (2) neo‐adjuvant chemotherapy prior to surgery; or (3) a past history of breast cancer. The protocol for the present study was approved by the Ethics Committee of FUSCC and the research is being reported in line with the STROBE guideline.

### Breast biopsy and surgery

We identified four biopsy methods to diagnose DCIS: fine needle aspiration, core needle biopsy, Mammotome biopsy, and open biopsy. Mammotome biopsy and paraffin sectioning of an open biopsy were categorized as “excision biopsies” in the analysis because both methods enabled full pathological evaluations while the others did not.

Patients were categorized according to the axillary evaluation received as follows: no evaluation, SLNB, or ALND. All SLNBs were performed at the same time as the breast surgeries. Touch imprint cytology was used to evaluate the SLN status intraoperatively, while the histological assessment with hematoxylin‐eosin staining performed postoperatively served as the golden standard. Level I and level II ALND was performed according to a standard ALND procedure.

### Statistical analysis

The clinicopathological variables were compared between the axillary evaluation group and the non‐evaluation group using Pearson's *χ* ^2^ test for categorical variables. Univariate and multivariate logistic regression analyses were performed to investigate the predictive value of the variables for axillary evaluation. Two‐tailed *P* values were adopted, and *P *<* *0.05 was considered significant. All statistical analyses were performed using SPSS version 20.0 (IBM, Chicago, IL, USA).

## Results

### Baseline characteristics

In total, 315 patients were included in the study. The average age was 47.5 ± 12.0 years, and 216 (68.6%) of the women were pre‐menopausal. At the initial presentation of DCIS, 222 (70.5%) patients presented with breast lumps, with an average diameter of 1.9 ± 0.9 cm. The majority of the patients (214, 67.9%) had undergone magnetic resonance imaging (MRI) prior to the final surgery.

Compared with the patients in the non‐evaluation group, patients in the axillary evaluation group were more likely to present with a lump upon physical examination (78.7% *vs*. 63.0%, *P *=* *0.009), to have undergone pre‐operative MRI (75.3% *vs*. 61.2%, *P *=* *0.007), to have less excision biopsy (55.3% *vs*. 73.3%, *P *=* *0.001), to have DCIS >1 cm according to the final pathology (36.0% *vs*. 12.7%, *P *<* *0.001), and to have more high‐grade histology cases and fewer low‐grade histology cases (*P *<* *0.001). The two groups were comparable with respect to estrogen receptor (*P *=* *0.562) and progesterone receptor (*P *=* *0.798) status, whereas they differed in the HER2 expression profile (*P *=* *0.004) (Table 1).

**Table 1:** Baseline clinicopathological characteristics of patients according to axillary evaluation status

Variables	Total*N* = 315	%	Axillary evaluation*N* = 150	%	No axillary evaluation*N* = 165	%	*P*‐value
Age							
≤50	205	65.1	105	70.0	100	60.6	0.081
>50	110	34.9	45	30.0	65	39.4	
Menopause							
No	216	68.6	110	73.3	106	64.2	0.057
Yes	95	30.2	40	26.7	55	33.3	
Unknown	4	1.3	0	0.0	4	2.4	
BC family history							
No	292	92.7	137	91.3	155	93.9	0.375
Yes	23	7.3	13	8.7	10	6.1	
Benign breast disease history							
No	289	91.7	137	91.3	152	92.1	0.800
Yes	26	8.3	13	8.7	13	7.9	
Lump							
Yes	222	70.5	118	78.7	104	63.0	**0.009**
No	89	28.3	31	20.7	58	35.2	
Unknown	4	1.3	1	0.7	3	1.8	
Lump size on PE							
<2 cm	81	25.7	42	28.0	39	23.6	0.100
≥2 cm	80	25.4	44	29.3	36	21.8	
Unknown	154	48.9	64	42.7	90	54.5	
Quadrant							
Upper outer	128	40.6	68	45.3	60	36.4	0.221
Others	173	54.9	77	51.3	96	58.2	
Unknown	14	4.4	5	3.3	9	5.5	
MRI							
Yes	214	67.9	113	75.3	101	61.2	**0.007**
No	101	32.1	37	24.7	64	38.8	
Excision biopsy							
Yes	204	64.8	83	55.3	121	73.3	**0.001**
No	111	35.2	67	44.7	44	26.7	
Tumor size on pathology							
≤1 cm	103	32.7	39	26.0	64	38.8	**<0.001**
>1 cm	75	23.8	54	36.0	21	12.7	
Unknown	137	43.5	57	38.0	80	48.5	
Grade							
Low	90	28.6	30	20.0	60	36.4	**0.001**
Median	122	38.7	61	40.7	61	37.0	
High	52	16.5	36	24.0	16	9.7	
Unknown	51	16.2	23	15.3	28	17.0	
ER							
Positive	247	78.4	116	77.3	131	79.4	0.562
Negative	38	12.1	21	14.0	17	10.3	
Unknown	30	9.5	13	8.7	17	10.3	
PR							
Positive	237	75.2	110	73.3	127	77.0	0.698
Negative	51	16.2	27	18.0	24	14.5	
Unknown	27	8.6	13	8.7	14	8.5	
HER2							
Negative	73	23.2	24	16.0	49	29.7	**0.004**
1+	95	30.2	43	28.7	52	31.5	
2+	67	21.3	35	23.3	32	19.4	
3+	45	14.3	31	20.7	14	8.5	
Unknown	35	11.1	17	11.3	18	10.9	

BC, breast cancer; PE, physical examination; MRI, magnetic resonance imaging; ER, estrogen receptor; PR, progesterone receptor; HER2, human epidermal growth factor receptor 2.

### Trends in axillary evaluation

Of the 315 patients, 135 (42.9%) underwent SLNB and 15 (4.8%) underwent ALND. No patients had lymph node metastasis based on the final paraffin section pathology. Among the patients who underwent SLNB, the median number of sentinel lymph nodes resected was 3 (range: 1–10). Intraoperative touch imprint cytology revealed 100% accuracy compared with the final pathology. Among the patients who underwent ALND, the median number of lymph nodes resected was 15 (range: 7–30).

**Figure 1:**
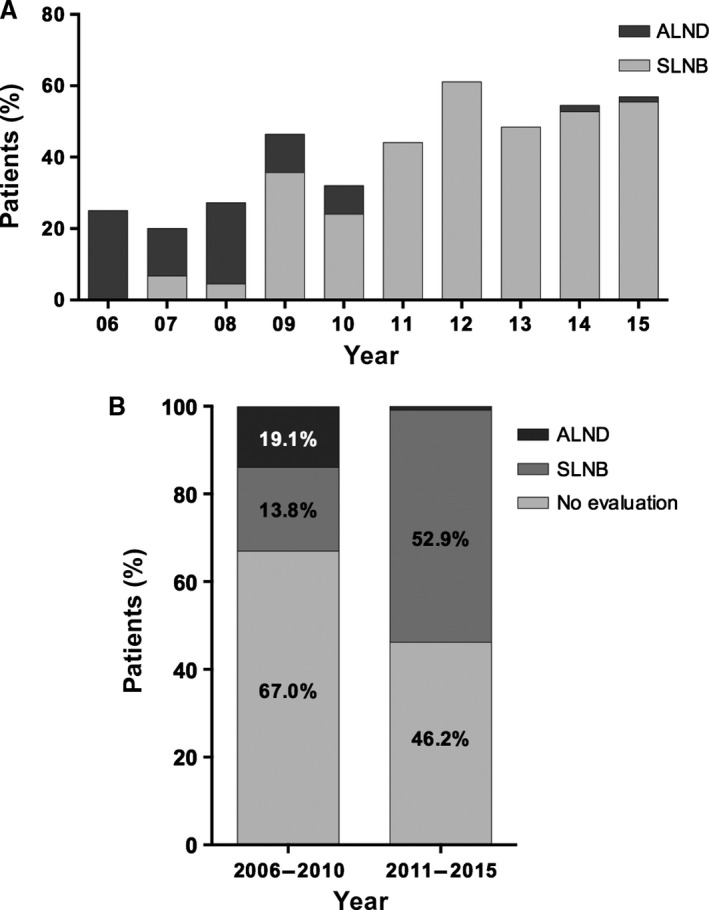
Trends in axillary evaluation in FUSCC. A, the proportion of patients who underwent axillary lymph node dissection (ALND) and sentinel lymph node biopsy (SLNB) from 2006 to 2015. B, the percentages of patients who underwent ALND, SLNB, and no axillary lymph node evaluation in 2006–2010 and 2011–2015.

The paradigm of axillary evaluation has changed over time (Table 2). Before 2008, fewer than 30% of patients underwent axillary evaluation; however, from 2009 to 2015, the percentile increased from 32.0% to 61.1%. SLNB was introduced at our institution in 2006 and was performed by only a few surgeons from 2006 to 2010 (no more than 10 cases each year). Therefore, 13 out of 15 ALNDs were performed during 2006–2010. Due to the high prevalence of SLNB in 2011–2015, less than 2% of patients underwent ALND each year (Fig. 1A).

**Table 2:** Trends in axillary evaluation from 2006 to 2015 in FUSCC

Year	Total	Axillary evaluation	%	SLNB	%	ALND	%
2006	4	1	25.0	0	0.0	1	25.0
2007	15	3	20.0	1	6.7	2	13.3
2008	22	6	27.3	1	4.5	5	22.7
2009	28	13	46.4	10	35.7	3	10.7
2010	25	8	32.0	6	24.0	2	8.0
2011	34	15	44.1	15	44.1	0	0.0
2012	36	22	61.1	22	61.1	0	0.0
2013	31	15	48.4	15	48.4	0	0.0
2014	55	30	54.5	29	52.7	1	1.8
2015	65	37	56.9	36	55.4	1	1.5
Total	315	150	47.6	135	42.9	15	4.8

SLNB, sentinel lymph node biopsy; ALND, axillary lymph node dissection.

Because of the increase in the prevalence of SLNB, we divided the whole cohort according to time periods: 2006–2010 and 2011–2015. Surprisingly, the proportion of patients who underwent axillary evaluation increased from 33.0% in 2006–2010 to 53.8% in 2011–2015, despite an increase in SLNB (from 13.8% to 52.9%) and a decrease in ALND (from 19.1% to 0.9%) (Fig. 1B). A change in axillary evaluation patterns was detected between the two periods (*P *<* *0.001).

### Predictors of axillary evaluation

**Figure 2:**
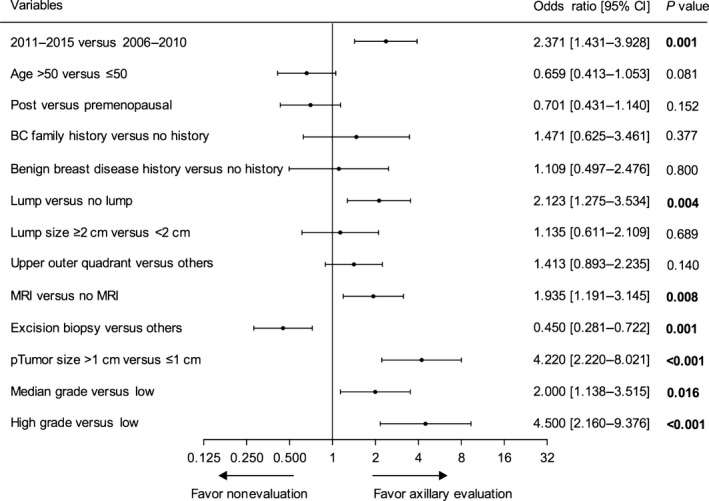
Univariate logistic regression analysis of predictive factors for axillary lymph node evaluation.

In the univariate logistic regression model, as expected, patients treated in 2011–2015 were more likely to have undergone axillary evaluation (odds ratio [OR] = 2.371, 95% confidence interval [CI]: 1.431–3.928, *P *=* *0.001) than those treated in 2006–2010. Palpable breast lumps on physical examination, receiving pre‐operative MRI, tumor size >1 cm on final pathology, median‐grade tumor, and high‐grade tumor were all predictive factors of axillary evaluation. However, patients diagnosed by excision biopsy were less likely to have axillary lymph evaluation than those diagnosed by other biopsy methods (Fig. 2).

**Figure 3:**
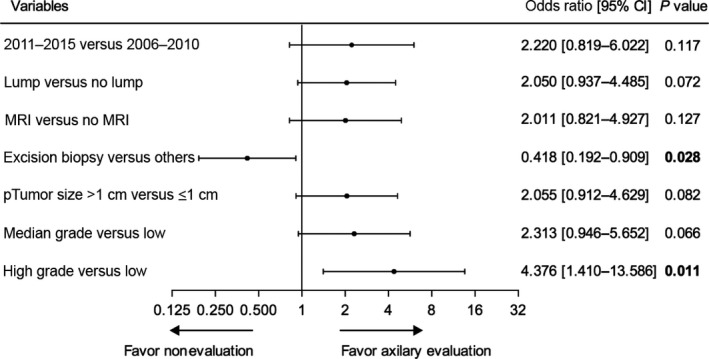
Multivariate logistic regression analysis of predictive factors for axillary lymph node evaluation.

All factors with *P *<* *0.05 in the univariate analysis were included in a multivariate logistic regression model. Patients with high‐grade tumor had a 4.376‐fold greater risk of undergoing lymph node evaluation than those with low‐grade tumor (95% CI: 1.410–13.586, *P *=* *0.011). Patients diagnosed by excision biopsy were 0.418‐fold less likely to have axillary lymph node evaluation than patients diagnosed by other biopsy methods (95% CI: 0.192–0.909, *P *=* *0.028) (Fig. 3).

**Figure 4:**
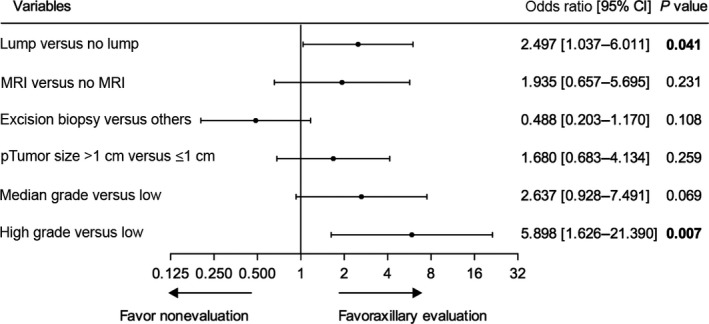
Multivariate logistic regression analysis of predictive factors for axillary lymph node evaluation in 2011–2015.

To eliminate the impact of the prevalence of SLNB, we confined our analysis to the period of 2011–2015, when SLNB was routinely adopted by all surgeons (*N* = 221). Multivariate analysis revealed that high‐grade tumor (OR = 5.898, 95% CI: 1.626–21.390, *P *=* *0.007) and palpable breast lump (OR = 2.497, 95% CI: 1.037–6.011, *P *=* *0.041) were independent predictors of axillary lymph node evaluation. Although significant in the previous univariate analysis, pre‐operative MRI, tumor size >1 cm on final pathology, and medium‐grade tumor were not predictive of axillary evaluation (Fig. 4).

## Discussion

In the current study, we identified a significant change in the paradigm of axillary evaluation in pure DCIS patients treated with BCT at our institute. Although SLNB replaced ALND as the main strategy, the overall axillary evaluation rate was as high as 47.6% among pure DCIS patients treated with BCT (42.9% SLNB; 4.8% ALND), much higher than the rate reported in previous studies. Coromilas et al. reported a 17.7% axillary evaluation rate among patients undergoing lumpectomy in 2006–2012, with 16.7% of patients undergoing SLNB and only 1.0% of patients undergoing ALND 11. Van Roozendaal et al. reported a 38.8% SLNB rate among core needle‐biopsied DCIS patients treated with BCT in the Netherlands in 2004–2013, while none of the patients underwent ALND 5. Worni et al. observed that for patients undergoing lumpectomy, the rate of SLNB increased from 1.4% to 17.8% and the rate of ALND decreased from 14.2% to 2.8% based on the National Cancer Institute's Surveillance, Epidemiology, and End Results (SEER) database from 1991 to 2010 12. Although Miller et al. also noticed an increasing trend in axillary evaluation from 2004 to 2013 for BCT patients, they reported a 22.6% rate of axillary surgery in DCIS patients treated with lumpectomy 6.

Despite the relatively high rate of axillary evaluation, we identified no lymph node metastasis in 150 DCIS patients who underwent axillary surgery. A recent study showed that 2% of patients with pure DCIS based on the final pathology were found to have SLN metastases, which the authors attributed to occult invasion 5. However, they included both BCT and mastectomy patients, whereas we limited our cohort to BCT patients, in whom the probability of missing occult invasion might be lower. Other studies showed SLN positivity rates ranging from 0.0 to 3.4% among patients with a final pathology of DCIS. The differences in these rates might be due to differences in sampling methods, serial sectioning, and immunohistochemical analysis 2, 3, 13, 14, 15, 16. These results justified the omission of axillary evaluation in DCIS patients.

However, there are still several circumstances under which axillary evaluation may be considered in DCIS patients treated with BCT. Our results demonstrated that a high‐grade tumor was an independent predictor of axillary evaluation in the overall cohort and the 2011–2015 subgroup and that a palpable breast lump was an independent predictor of axillary evaluation in the 2011–2015 cohort. These results were consistent with guideline recommendations and previous studies reporting that a palpable tumor and high‐grade tumor indicated an increased risk of harboring an occult malignancy, therefore, supporting axillary evaluation as an option in these cases 6, 7, 15, 16, 17. Another reported clinicopathological risk factor for axillary evaluation in DCIS was large tumor size 7, 15; however, this factor was not significant in our cohort. A possible explanation is that only highly selected patients with a small tumor size received BCT in our cohort. We found that only 23.8% patients had a tumor >1 cm according to the final pathology, with the tumor size remaining unknown in 43.5% of patients.

We also identified biopsy method as an independent predictor of axillary evaluation. Unlike previous studies, up to 64.8% patients had excision biopsy in our cohort. Open biopsy and Mammotome biopsy could potentially provide adequate tissue for pathology examination and decrease the chance of upstaging to invasive disease in the second surgery; therefore, they were associated with a lower incidence of axially evaluation. By contrast, DCIS diagnosed by core needle biopsy had an approximately 20% chance of being upstaged to invasive cancer due to the inadequate sampling 5, 18. Even in core needle biopsy‐diagnosed DCIS, a smaller core needle size was also positively correlated with an increased rate of upstaging and SLN metastases 5. Future studies should investigate the optimal biopsy method for clinically suspicious DCIS and decrease unnecessary axillary evaluation due to inadequate sampling.

Miller et al. also observed that ER negativity was a predictive factor of both SLNB and tumor upstaging in DCIS patients 6. Because the immunohistochemistry results were not available before the final surgery at our institute, ER, PR, and HER2 status were not considered in the decision to perform axially evaluation; therefore, we did not include these factors in the logistic regression model. Furthermore, a recent study indicated that a high volume of procedures performed by the surgeon was a significant predictor of axillary evaluation omission in DCIS patients undergoing BCT, which highlighted the importance of physician education and experience 11.

Interestingly, the increased rate of axillary evaluation was correlated with the prevalence of SLNB at our institute. More patients underwent axillary evaluation in 2011–2015 than in 2006–2010 (33.0% *vs*. 53.8%). The overuse of SLNB could be explained by the following hypothesis. First, 70.5% of the patients in our cohort presented with a breast lump, indicating a high possibility of harboring invasive disease. Second, from the patients’ perspective, there was a low acceptance of longer waiting times for definitive surgery and a reluctance to return to the operating room if invasive cancer were identified. Finally, from the surgeons’ perspective, SLNB was embraced as a less traumatic surgery than ALND and could eliminate the need for a second operation if invasive cancer were found on final pathology. However, although the complication rate decreased by three‐fold in SLNB compared with ALND, the comorbidity of SLNB could still be underestimated 19. Multiple studies have found lymphedema in 7–8% of patients treated with SLNB alone at 6 months 19, 20, 21 and 8.2–15% at 1 to 2 years 9, 22, 23, whereas post‐ALND lymphedema was diagnosed in 11–14% of patients 19, 20.

There are some limitations in this current study. First, this was a retrospective study. However, this was a reliable dataset with uniform inclusive and exclusive criteria. Second, this was a single center study. While, the data reported in this current study was comparable with previous studies, supporting omitting axillary evaluation in DCIS patients treated with BCT. Further assessment is needed to select patients with low risk of axillary metastasis preoperatively, who can safely omit axillary evaluation.

## Conclusions

In sum, the present study revealed both a significant decrease in ALND in pure DCIS patients treated with BCT overtime and a concerning overuse of SLNB at our institute. Despite the relatively high rate of axillary evaluation, we identified no axillary lymph node metastasis, which justified omitting axillary evaluation in these patients. High‐grade tumor, palpable lump, and biopsy method were recognized as independent predictors of axillary evaluations. Excision biopsy of suspicious DCIS lesions may potentially preclude the invasive component of the disease and might help to avoid axillary surgery.

## Conflicts of Interest

None.
